# Adverse Respiratory Reactions to Tyrosine Kinase Inhibitors: A Disproportionality Analysis of Spontaneous Reports from European Countries

**DOI:** 10.3390/life16010113

**Published:** 2026-01-13

**Authors:** Ilaria Ammendolia, Carmen Mannucci, Emanuela Esposito, Gioacchino Calapai, Mariaconcetta Currò, Paola Midiri, Cristina Mondello, Antonino Cancellieri, Luigi Cardia, Fabrizio Calapai

**Affiliations:** 1 Department of Chemical, Biological, Pharmaceutical and Environmental Sciences, University of Messina, 98166 Messina, Italy; ilaria.ammendolia@unime.it (I.A.); emanuela.esposito@unime.it (E.E.); 2 Department of Biomedical, Dental Sciences and Morphological and Functional Imaging, University of Messina, 98125 Messina, Italy; carmen.mannucci@unime.it (C.M.); cristina.mondello@unime.it (C.M.); fabrizio.calapai@unime.it (F.C.); 3 Department of Clinical and Experimental Medicine, University of Messina, 98125 Messina, Italy; mariaconcetta.curro@unime.it (M.C.); paola.midiri@polime.it (P.M.); 4 Department of Human Pathology of Adult and Childhood “Gaetano Barresi”, University of Messina, 98125 Messina, Italy; antonino.cancellieri@unime.it (A.C.); luigi.cardia@unime.it (L.C.)

**Keywords:** tyrosine kinase inhibitors, dasatinib, bosutinib, asciminib, imatinib, nilotinib, ponatinib, chronic myelogenous leukemia, pleural effusion, pulmonary arterial hypertension

## Abstract

Background: The tyrosine kinase inhibitors (TKIs) asciminib, bosutinib, dasatinib, imatinib, nilotinib, and ponatinib have been approved for chronic myelogenous leukemia (CML) therapy. However, pharmacovigilance reports associated with these drugs are neither consistent nor homogenous, with reports of pulmonary toxicity, which could limit their utilization. To better clarify TKIs’ pulmonary risk, we used the European database EudraVigilance to conduct a study on adverse events suspected to be caused by the TKIs asciminib, bosutinib, dasatinib, imatinib, nilotinib, and ponatinib when used for CML therapy. Methods: Suspected adverse reactions to TKIs in the EudraVigilance database (2020–2024) coming from European countries and the United Kingdom were analyzed and compared through a disproportionality analysis. Results: The most frequent alerts concerned the respiratory disorders “pleural effusion” (PE) and “pulmonary arterial hypertension” (PAH) in relation to dasatinib and bosutinib use. Among the TKIs, the prescription of dasatinib is associated with a higher occurrence of PE and PAH, while the prescription of bosutinib induces PE at a minor frequency that nonetheless carries a significant risk for PAH, occurring more often in women. Conclusions: The results indicate that respiratory disorders induced by the TKIs dasatinib and bosutinib need to be diagnosed in a timely manner, and suggest that caution should be taken when prescribing these TKIs to patients affected by CML and pulmonary comorbidities.

## 1. Introduction

Chronic myelogenous leukemia (CML) is a disease that involves myeloproliferation at the Philadelphia chromosome t(9;22) (q34;q11) and/or the breakpoint cluster region–Abelson 1 (BCR-ABL1) fusion gene, a genetic abnormality representing a hallmark of CML and other leukemias. t(9;22) (q34;q11) translocation induces the genesis of the Philadelphia (Ph) chromosome and the BCR-ABL1 fusion gene. This generates a constitutively active tyrosine kinase, driving proliferation, blocking apoptosis, and causing genetic unreliability, crucial hallmarks of CML [1]. This cytogenetic disorder is characterized by the expression of the BCR-ABL1 protein activated by tyrosine kinase (TK) [2]. The annual incidence of CML is approximately 2 cases per 100,000 people [3]. Symptomatology is variable: commonly, many patients have nonspecific symptoms while, for about 40% of patients, diagnosis occurs accidentally following routine laboratory examinations, revealing inexplicable leukocytosis. The most frequent symptom is splenomegaly, which is evident in around 50% of newly diagnosed subjects [4]. If not treated, CML generally progresses from a chronic to accelerated phase and, eventually, to blast crisis—a phase that mimics acute leukemia [5]. CML progression is, for the most part, due to the genetic instability transmitted by BCR-ABL1, which facilitates the acquisition of further cytogenetic and molecular abnormalities driving the transition from the chronic to accelerated phases and then to blast crisis [6]. In the past, recombinant interferon-alfa, cytarabine, and hematopoietic cell transplantation were used to treat Philadelphia chromosome/BCR-ABL1 CML; however, drugs that act through the inhibition of tyrosine kinase (TK) activity are used now, with the tyrosine kinase inhibitors (TKIs) asciminib, bosutinib, dasatinib, imatinib, nilotinib, and ponatinib having been approved for CML therapy [7].

A recent pharmacovigilance study based on adverse events to TKIs signaled in oncologic patients used the Food and Drug Administration Adverse Event Reporting System (FAERS) database to report that drugs linked to BCR-ABL1 TKIs reveal a safety profile that is not consistent or homogenous [8]. Although considered effective in terms of clinical response, the TKI dasatinib has been associated with potential pulmonary toxicity characterized by pulmonary arterial hypertension (PAH) and pleural effusion (PE), to the point that its utilization is limited [9]. These adverse events occur at the beginning of therapy and, in general, are transitory even if special attention is needed (particularly in high-risk patients) [10]. Furthermore, a disproportionality analysis showed that dasatinib, bosutinib, ponatinib, and nilotinib exhibit significant disproportionality signals for PAH induced by PKIs [11]. Cases of bosutinib-induced lung injury potentially causing life-threatening complications have also been reported, suggesting that patients treated with bosutinib should be carefully monitored for pulmonary safety [12].

With the aim of providing a clearer definition of TKI safety and the pulmonary risk associated with these drugs, we used the European database EudraVigilance to conduct a study on adverse respiratory events suspected to be caused by TKIs used for CML. Descriptive and disproportionality analyses were performed on reports of adverse reactions associated with the TKIs dasatinib, asciminib, bosutinib, imatinib, nilotinib, and ponatinib when used for CML and signaled in the years 2020–2025 in the European Union (EU) and the United Kingdom (UK).

## 2. Materials and Methods

EudraVigilance is a databank that collects cases of suspected adverse reactions (SARs) linked to medicines approved for use in the European Union (EU), with SARs detected by the national drug regulatory authorities of the EU or by drug companies reported in terms of individual cases (Individual Case Safety Reports; ICSRs) [13]. EudraVigilance gathers signals of “suspected” adverse reactions, defined as undesired medical events that occur following the use of medicinal products but are not necessarily linked to or induced by the drug itself [14].

### 2.1. Design of This Study

ICSRs reporting SARs that occurred following the prescription of dasatinib, imatinib, nilotinib, ponatinib, bosutinib, and asciminib from 1 January 2020 to 31 December 2024 were collected, analyzed, and compared. All the drugs were examined throughout the study period from their respective launch dates. Data on SARs was collected from the public database EudraVigilance based on the following inclusion criteria: serious and non-serious SARs, and reports from healthcare professionals of cases concerning all ages (from 0 to >85 years) in the European Economic Area (EEA), including the UK. Although the UK is no longer a part of the European Union, it is included in this analysis because it is incorporated in the EEA in the EudraVigilance database. In EudraVigilance, SARs are classified as serious using pharmacovigilance criteria, focusing on the seriousness of the events: it resulted in death, it was life-threatening, it resulted in or prolonged hospitalization, it resulted in disability, or it was related to congenital anomalies [15]. Signals were excluded if they were sent by non-healthcare professionals or from non-European countries. For each case, patient characteristics (age group and sex), adverse reactions (often more than one for each ICSR), and primary sources were supplied. Regarding the criteria for data extraction, SAR selection was based on the Medical Dictionary for Regulatory Activities (MedDRA) [16]. The MedDRA was used to code cases in pharmacovigilance databases reporting adverse events and to ease the search for adverse drug reactions. Each SAR was analyzed for a single case. Single SAR events were gathered following the SOC (System Organ Classification) as described in the MedDRA hierarchy, for example, “skin and subcutaneous tissue disorders”, “cardiac disorders”, or “vascular disorders”. SOC, a method to cluster medical terms according to body systems or functions, constitutes the upper level in the hierarchy because it encompasses the largest concept used for data recovery. The terms “pleural effusion” and “pulmonary arterial hypertension” were used as the “preferred term” (PT) cataloged in the MedDRA. A PT is a definite descriptor for an adverse reaction. All cases reporting adverse events signaled individually in the ICSR as “pleural effusion” and/or “pulmonary arterial hypertension” for dasatinib, imatinib, nilotinib, ponatinib, bosutinib, and asciminib were selected, calculated, and analyzed for their frequency.

### 2.2. Data Analysis

The data extraction source was a table where each row shows an ICSR and columns contain a distinct data point associated with the case. The data were analyzed by grouping the PTs of single cases with an upper level of the MedDRA hierarchy by unifying individual serious SARs in the SOC level. A serious/non-serious (S/NS) ratio was calculated for each drug. Only cases classified as serious were analyzed. In accordance with the E2D guidelines of the International Council for Harmonization, ICSRs were classified as serious if they were life-threatening, resulted in death, resulted in or prolonged hospitalization or disability, or were related to a congenital anomaly/birth defect or other medically important conditions. Adequate stratification of alerts by sex and age groups was performed to avoid biases caused by confounding effects and to analyze these two variables separately. The sex distribution was analyzed using a chi-square test. The significance level was set at *p* < 0.05. Duplicate and incomplete ICSRs were excluded from the analysis. A duplicate search was conducted on the dataset based on similarity in terms of adverse reactions, age, sex, suspected/interacting medicinal products, and EudraVigilance local report number. The potential association of the SOC group “respiratory, thoracic, and mediastinal disorders” was analyzed for disproportionality based on reporting odds ratios (RORs) and a comparison of the SARs of dasatinib or bosutinib with those signaled for the other TKIs. Disproportionality analysis—a methodology used to detect adverse drug reaction alerts—is used to compare the proportions or frequencies of two or more groups and to verify whether the differences are statistically significant based on a two-by-two contingency table. In this way, the differences between the occurrence and background frequencies for target drugs and target adverse events can be compared [17]. The ROR is calculated as the odds ratio for a selected drug versus that of other drugs for a certain adverse event [18]. It is used here to establish the strength of disproportionality by comparing SARs signaled for TKIs. In particular, an ROR of 1 indicates the absence of any alerts; conversely, an ROR greater than 1 indicates an alert and the existence of an association [19]. All statistical analyses were performed using the SPSS statistical software, version 29.0 (SPSS, IBM, Armonk, NY, USA).

## 3. Results

### 3.1. Percentage and Ratio of Serious/Non-Serious Suspected Adverse Reactions of the TKIs Dasatinib, Imatinib, Nilotinib, Ponatinib, Bosutinib, and Asciminib

Table 1 shows the total number of ICSRs, the number of serious and non-serious cases, and the serious/non-serious ratio of SAR signals related to dasatinib, asciminib, bosutinib, imatinib, nilotinib, and ponatinib prescription. The S/NS ratio of all the drugs is more than 1. The highest ratio is found for dasatinib prescription (Table 1).

### 3.2. Reporting Odds Ratio (ROR) of Serious Suspected Adverse Reactions to Dasatinib, Asciminib, Bosutinib, Imatinib, Nilotinib, and Ponatinib According to Their System Organ Classification (SOC)

The RORs and confidence intervals of SARs are reported in Table 2, grouped according to their SOC. For each drug, differences were found in their safety profiles with respect to that for all examined TKIs: Cardiac and vascular disorders are more often signaled with the use of nilotinib and ponatinib. Gastrointestinal disorders are more frequently reported with bosutinib prescription. More general disorders are signaled with the use of imatinib, nilotinib, and dasatinib. Injury, poisoning, and procedural complications are associated with the use of ponatinib, while infections and infestations with the use of asciminib. Investigations and neoplasms are more frequently reported with nilotinib and ponatinib prescription, respectively, as are nervous system disorders. By contrast, imatinib use was more frequently associated with skin and subcutaneous disorders. However, the most significant data is undoubtedly the dramatic increase in reporting of respiratory, thoracic, and mediastinal disorders associated with dasatinib use. This group of disorders was reported to have increased also with bosutinib use, but to a much lesser extent.

### 3.3. Sex Distribution of Serious Suspected Adverse Reactions “Pleural Effusion” and “Pulmonary Arterial Hypertension” Associated with Dasatinib and Bosutinib Prescription

The sex distribution of signals reporting the adverse reaction PE in association with dasatinib prescription shows an apparent prevalence in males, 55.7%. However, statistical analysis does not confirm this finding: “pulmonary arterial hypertension” is equally reported between the sexes with dasatinib use. However, an asymmetric sex distribution is observed following bosutinib prescription. PE is more often signaled for males, while PAH for females.

PE is reported in 32.5% of all serious cases associated with dasatinib and in 5.16% for bosutinib. “Pulmonary arterial hypertension” is reported in 4.13% and 3.29% of all serious cases signaled for dasatinib and bosutinib, respectively (Table 3). The age distribution shows that PE is reported more often in elderly patients (65–85 years), both for dasatinib and bosutinib. PAH is more often signaled in adults (18–64 years) with dasatinib and in elderly patients (65–85 years) with bosutinib (Table 4).

### 3.4. Reporting Odds Ratio (ROR) of Individual Case Safety Reports (ICRSs) Associated with Dasatinib and Bosutinib Prescription, Reporting “Pleural Effusion” and “Pulmonary Arterial Hypertension” Compared with the Tyrosine Kinase Inhibitors Asciminib, Imatinib, Nilotinib, and Ponatinib

A disproportionality analysis was carried out through the evaluation of RORs obtained by comparing the signals for “pleural effusion” and “pulmonary arterial hypertension” related to dasatinib and bosutinib prescription. Data for the two drugs were compared separately with those for the other TKIs. The results show RORs of 9.55 (95%; C.I. 7.28–12.52) for PE suspected to be caused by dasatinib and 2.32 (95%; C.I. 1.61–3.36) for PE suspected to be caused by bosutinib. The RORs of PAH were 4.90 (95%; C.I. 2.98–8.05) for dasatinib prescription and 4.70 (95%; 2.68–8.25) for bosutinib prescription (Table 5).

## 4. Discussion

PE is a condition characterized by excess fluid in the pleural space. The pleural space is the gap between the lungs and the chest wall, which normally contains a small amount of fluid [20]. PAH is another respiratory disorder characterized by high blood pressure in the pulmonary arteries. This condition, characterized by constriction of small arteries, forces the right side of the heart to work harder, leading to potential heart failure [21]. These adverse reactions, requiring discontinuation of therapy and the initiation of an appropriate medical intervention [22], have been associated with the prescription of dasatinib [8]. The present analysis indicates that the use of the TKIs dasatinib and bosutinib, although in a different context, can expose patients to these respiratory disorders.

Dasatinib is a second-generation TKI, indicated as first-line pharmacological therapy for people with a new diagnosis of CML and as second-line therapy for patients who show resistance or intolerance to treatment with imatinib [23]. However, despite the superior clinical effectiveness of dasatinib versus imatinib, its potential pulmonary toxicity may limit its clinical use [10]. PE, affecting 14% to 35% of patients prescribed dasatinib, seems to be caused by an imbalance in oncotic and hydrostatic pressures, increased capillary permeability, or impaired lymphatic drainage [24].

Dasatinib frequently causes lymphocytosis in people affected by CML. This hematologic picture is generally characterized by the presence of large granular lymphocytes (LGLs), seemingly related to the efficacy of the drug and associated with the development of PE. No signals reporting LGLs or lymphocytosis were found in association with dasatinib prescription in the EudraVigilance database in the years 2020–2024. The reason for this failure in reporting could be due to the rapidity and transience of this effect, which, together with the fact that it is considered a positive prognostic sign indicating an improvement in CML [25], leads to the lack of consideration of LGL as an adverse effect. Bosutinib is a second-generation TKI indicated for the pharmacological treatment of subjects with Ph-positive chronic-, accelerated-, or blast-phase CML who are intolerant or resistant to other TKIs. Bosutinib inhibits a tyrosine kinase oncogene and Src kinases responsible for CML pathogenesis [26]. More recently, another case report described refractory recurrent PE caused by bosutinib in a 79-old-year woman. The event led to the development of worsening dyspnea related to respiratory toxicity in the form of PE [27].

An early analysis of adverse events using data from the FAERS database raised the hypothesis of an association between PE and PAH, and the prescription of dasatinib, even if these events were considered rare [28]. This concern has been raised specifically for PE with the publication of a case series reporting the association of chylothorax (a condition with chyle in the pleural space) [29] with dasatinib use, which required treatment through the administration of diuretics and steroids, reductions in dose, and discontinuation of the drug [30]. Different hypotheses have been proposed for the mechanism underlying chylothorax induced by dasatinib, such as immune factors, blocks in T-cell function, and inhibition of platelet-derived growth factor receptors expressed in pericytes involved in angio-lymphangiogenesis [31]. Moreover, a retrospective pharmacovigilance study on TKIs based on the FAERS database collecting signals reported from 1 January 2004 to 30 September 2021 and published in the year 2023 showed that people aged ≥ 65 years using dasatinib, imatinib, nilotinib, or bosutinib had more elevated RORs for PE, pericardial effusion, and pulmonary edema [32].

Pulmonary hypertension, a rare but potentially life-threatening complication, is described as an increase in mean pulmonary arterial pressure to > 25 mmHg [33]. The pathophysiological mechanism linking this adverse reaction to dasatinib remains unclear, but together with endothelial dysfunction and vascular remodeling, the inhibition of BCR-ABL and Src family kinases induced by the drug has been found to contribute to PAH development [34,35]. A disproportionality analysis performed in 2019 with data from the World Health Organization database VigiBase, through RORs and 95% confidence intervals, found a positive signal indicating associations between dasatinib bosutinib, ponatinib, ruxolitinib, and nilotinib and increases in the occurrence of pulmonary arterial hypertension. The authors found dasatinib’s safety profile to be dissimilar with respect to those of other drugs of the same category and suggested that kinases of the bone morphogenetic protein signaling pathway could also play a role in the development of PAH induced by TKIs [11].

Our analysis was conducted using the European database system EudraVigilance. This is the first time that a post-marketing pharmacovigilance study on potentially adverse reactions induced by TKIs has been designed using data derived exclusively from real-world European data through an analysis of the EudraVigilance database. The use of this database offers a European perspective and allows for the early identification and evaluation of potential safety issues (signals) for medicines in the EEA based on pharmacovigilance systems, which are homogenous among the different European countries and UK. The data from our study include a descriptive analysis and a disproportionality analysis about adverse reactions, particularly respiratory disorders induced by the TKIs dasatinib, asciminib, bosutinib, imatinib, nilotinib, and ponatinib. A calculation of the ratio between serious and non-serious adverse reactions of all the drugs gives a value of more than 1 for all examined drugs and shows that the highest ratio is linked to the prescription of dasatinib. A high S/NS ratio acts as a strong indicator of potential underlying toxicity, suggesting that these drugs carry a greater inherent risk for severe harm, even with fewer total reports, and that their safety profile warrants closer scrutiny [36].

An analysis of the SAR data signaled in the years 2020–2024, grouped according to their SOC level and examined in this study, shows a different profile among the TKIs. As expected, the most significant data obtained from the disproportionality analysis is the dramatic increase in reporting of respiratory, thoracic, and mediastinal disorders associated with the prescription of dasatinib (ROR 10.77; 95% C.I. 8.69–13.34). A closer look at the association of the respiratory disorders PE and PAH with dasatinib prescription shows that they are reported in 32.5% and 4.13% of all serious cases, respectively. This data confirms previous findings indicating that PE is more commonly signaled than PAH with the use of dasatinib. A recent study reported that 17.6% of patients treated with dasatinib developed PE [37], while, as a cause of PAH, the lowest estimated incidence based on French Pulmonary Hypertension registry data, is 0.45% [38]. In our study, an apparent asymmetric sex distribution, indicating a male prevalence between the patients treated with dasatinib in which these adverse reactions occur, has not been confirmed by statistical analysis. The age distribution shows PE being reported more often in elderly patients (65–85 years), while PAH in adults (18–64 years). The disproportionality analysis performed to compare the signals for PE and PAH in association with dasatinib and data on the same adverse reactions reported with the TKIs asciminib, bosutinib, imatinib, nilotinib, and ponatinib produced an ROR of 9.55 (95%; C.I. 7.28–12.52) for the adverse reaction PE and an ROR of 4.90 (95%; C.I. 2.98–8.05) for the adverse reaction PAH, thus indicating that this drug exhibits a strong signal for disproportionate reporting compared with the combined odds of those events with other drugs in the same category. This finding means that, in association with the prescription of dasatinib, compared with the odds the other TKIs, PE and PAH have been reported 9.55 and 4.90 times more often, respectively. This result indicates a strong safety signal for the adverse event PE and a lesser, but not negligible level, for PAH in association with dasatinib prescription.

The disproportionality analysis for respiratory, thoracic, and mediastinal disorders potentially induced by bosutinib shows an ROR of 2.38 (95%; C.I. 1.76–3.24) in comparison with the other TKIs (including dasatinib). Furthermore, evaluation of the ROR for both the respiratory disorders PE and PAH induced by bosutinib shows that these disorders are signaled in 5.16% and 3.29% of all serious cases induced by this drug, respectively. The incidence of PE following the use of bosutinib was reported as being largely variable, present within 1–10% of patients [39]. The sex distribution of European data shows an asymmetric difference between PE and PAH induced by bosutinib, indicating statistically significant prevalence of PE in males and of PAH in females. The disproportionality analysis comparing the signals for PE and PAH associated with bosutinib and the data for these adverse reactions reported with the TKIs dasatinib, asciminib, imatinib, nilotinib, and ponatinib produced RORs of 2.32 (95%; C.I. 1.61–3.36) and 4.70 (95%; C.I. 2.68–8.25), respectively. These data indicate that the use of bosutinib is correlated with increased reporting of PE and PAH, 2.32 and 4.70 higher than for the other TKIs. The ROR for PE indicates an association that warrants attention, although showing lower strength of association than the association between PE and dasatinib; however, the values for PAH are almost comparable for the two drugs. The sex distribution shows a difference characterized by a prevalence of male patients experiencing PE and a prevalence of females affected by PAH following bosutinib prescription. The distribution of pleural effusion has been described as overlapping between the sexes, although the higher overall incidence for males is sometimes due to cancer, induced by smoking [40]. Further research is therefore needed to better understand the differences in the frequency of SAR reporting between males and females as obtained from the EudraVigilance database. The difference in sex distribution of PAH can be explained by the greater susceptibility of women to PAH compared with men. Women have a greater adaptability of their right ventricle and thus better survival; however, alterations in estrogen synthesis and metabolism appear to be key factors in determining the observed differences in vulnerability to PAH between the sexes [41].

An ROR higher than 1, as observed in our analysis, means the odds for specific adverse events occurring with certain drugs is more elevated compared with the combined odds of these events (in this case, PE and PAH) signaled for other drugs in the same category (or all other drugs), indicating a strong signal for disproportionate reporting [42]. However, the results of our analysis have to be interpreted with caution due to the limitations of pharmacovigilance investigations conducted on databases of spontaneous signals for adverse drug effects. Such limitations include the arbitrary selection of years considered for analysis (2020–2024); the absence of a denominator; under-reporting; the lower quality of signals; causal relationship uncertainty; and, finally, difficulties managing confounding factors such as comorbidities or, sometimes, dosage and duration of exposure, which can influence health/pathology conditions. Despite the abovementioned limitations, these data provide an updated report on the occurrence of respiratory disorders following dasatinib use. They confirm that risk is dramatically elevated for dasatinib-induced PE, as well as dasatinib- and bosutinib-induced PAH in comparison with other TKIs as the disproportionality analysis indicates that the risk of PAH is similar for these two drugs but almost 5 times higher than that for the other TKIs.

## 5. Conclusions

In conclusion, this is the first post-marketing surveillance report conducted through an analysis of data exclusively coming from European countries and the United Kingdom. The results indicate that the prescription of dasatinib is accompanied by higher occurrence of PE and PAH compared with the prescription of other TKIs, while the prescription of bosutinib induces PE at a minor frequency that nonetheless carries a significant risk for PAH, occurring more often in women. These results indicate that respiratory disorders induced by the TKIs dasatinib and bosutinib need to be diagnosed in a timely manner and suggest that caution should be taken when prescribing TKIs to patients affected by CML.

## Figures and Tables

**Table 1:** Serious and non-serious Individual Case Safety Reports (ICSRs) related to the tyrosine kinase inhibitors dasatinib, asciminib, bosutinib, imatinib, nilotinib, and ponatinib, signaled in the years 2020–2024 in the European Economic Area and the United Kingdom.

Drug	ICSRs (Total)	Serious ICSRs	Non-Serious ICSRs	ICSRs Serious/Non-Serious Ratio
Dasatinib	703	508	195	2.60
Asciminib	125	79	46	1.72
Bosutinib	337	213	124	1.72
Imatinib	1773	1116	657	1.70
Nilotinib	604	413	191	2.16
Ponatinib	467	326	141	2.31

**Table 2:** Reporting odds ratio (ROR) of signals for serious suspected adverse reactions related to the prescription of the tyrosine kinase inhibitors dasatinib, asciminib, bosutinib, imatinib, nilotinib, ponatinib, signaled in the years 2020–2024, in the European Economic Area and United Kingdom, clustered according to System Organ Classification (SOC).

SOC	Imatinib	Nilotinib	Ponatinib	Dasatinib	Bosutinib	Asciminib
	Cases	Cases	Cases	Cases	Cases	Cases
	ROR	ROR	ROR	ROR	ROR	ROR
	95% C.I.	95% C.I.	95% C.I.	95% C.I.	95% C.I.	95% C.I.
**Blood and lymphatic system disorders**	208	80	54	93	31	15
	1.06	1.10	0.88	1.01	0.65	1.06
	(0.87–1.30)	(0.84–1.44)	(0.65–1.21)	(0.79–1.30)	(0.44–0.97)	(0.60–1.88)
**Cardiac disorders**	109	85	68	80	34	15
	0.48	2.77	1.94	1.40	1.21	1.37
	(0.38–0.61)	(2.10–3.65)	(1.45–2.60)	(0.07–1.82)	(0.83–1.78)	(0.77–2.43)
**Gastrointestinal disorders**	273	57	57	103	52	12
	1.45	0.91	1.15	1.42	1.58	0.80
	(1.20–1.75)	(0.68–1.23)	(0.85–1.55)	(0.12–1.80)	(1.14–2.19)	(0.43–1.49)
**General disorders and administration site conditions**	393	118	73	130	43	21
	2.20	2.26	1.17	1.61	0.99	1.35
	(1.84–2.63)	(1.79–2.85)	(0.89–1.54)	(1.29–2.00)	(0.70–1.39)	(0.81–2.24)
**Injury, poisoning, and procedural complications**	92	23	50	35	8	2
	1.10	0.52	2.23	0.88	0.39	0.25
	(0.83–1.47)	(0.33–0.81)	(1.59–3.14)	(0.60–1.28)	(0.19–0.81)	(0.06–1.04)
**Infections and infestations**	71	23	21	46	13	14
	0.86	0.59	0.77	1.43	0.75	2.49
	(0.64–1.17)	(0.38–0.93)	(0.48–1.24)	(1.01–2.03)	(0.42–1.33)	(1.37–4.53)
**Investigations**	136	82	30	47	25	12
	0.95	2.17	0.75	0.80	0.96	1.23
	(0.75–1.20)	(1.65–2.86)	(0.50–1.11)	(0.58–1.10)	(0.62–1.48)	(0.66–2.30)
**Benign, malignant, and unspecified neoplasms**	133	44	46	41	12	14
	1.19	0.98	1.45	0.77	0.46	1.68
	(0.93–1.52)	(0.70–1.38)	(1.03–2.04)	(0.54–1.09)	(0.25–0.84)	(0.93–3.04)
**Nervous system disorders**	109	80	57	41	16	11
	0.70	2.19	1.82	0.71	0.59	1.16
	(0.55–0.90)	(1.66–2.90)	(1.33–2.48)	(0.50–1.00)	(0.35–1.00)	(0.61–2.22)
**Respiratory, thoracic, and mediastinal disorders**	125	32	42	267	67	8
	0.34	0.46	0.79	10.77	2.38	0.51
	(0.27–0.42)	(0.31–0.66)	(0.56–1.10)	(8.69–13.34)	(1.76–3.24)	(0.24–1.06)
**Skin and subcutaneous disorders**	175	44	31	39	33	10
	1.63	0.89	0.78	0.63	1.36	0.99
	(1.30–2.06)	(0.64–1.25)	(0.53–1.14)	(0.44–0.89)	(0.92–2.01)	(0.50–1.94)
**Vascular disorders**	54	60	37	25	9	11
	0.50	2.20	1.58	0.63	0.49	1.80
	(0.36–0.69)	(1.59–3.05)	(1.08–2.30)	(0.41–0.97)	(0.24–0.96)	(0.94–3.47)

RORs and confidence intervals (C.I.; in brackets) are reported as the result of comparisons between each drug.

**Table 3:** The sex distribution of serious suspected adverse reactions to the drugs dasatinib and bosutinib, signaled in the years 2020–2024, with the preferred terms “pleural effusion” and “pulmonary arterial hypertension” in the European Economic Area and the United Kingdom. The total number of Individual Case Safety Reports (ICRSs) of dasatinib and bosutinib is 508 and 2013, respectively. The total number of serious male and female cases signaled for dasatinib is 267 and 241, respectively, and for bosutinib is 116 and 97, respectively.

Drug	Adverse Reaction	Male Cases Number and %	Female Cases Number and %	Male and Female Cases	% of All Serious ICSRs	Sex Distribution Significance Level (*p*)
Dasatinib	Pleural effusion	92 (55.7%)	73 (44.3%)	165	32.5%	N.S. 0.3167
	Pulmonary arterial hypertension	17 (50.0%)	17 (50.0%)	34	4.13%	N.S. 0.7570
Bosutinib	Pleural effusion	28 (68.3%)	13 (31.7%)	41	5.16%	0.0478 *
	Pulmonary arterial hypertension	4 (22.2%)	14 (77.8%)	18	3.29%	0.0041 *

* *p* = <0.05 males vs. females. N.S. = not significant.

**Table 4:** Age distribution of serious suspected adverse reactions to the drugs dasatinib and bosutinib, signaled in the years 2020–2024, with the preferred terms “pleural effusion” and “pulmonary arterial hypertension” in the European Economic Area and the United Kingdom. Total number of Individual Case Safety Reports (ICRSs) for dasatinib = 508.

Drug	Adverse Reaction	18–64 (Years)	65–85 (Years)	>85 (Years)
Dasatinib	Pleural effusion	30 (5.9%)	41 (8.1%)	3 (0.6%)
	Pulmonary arterial hypertension	13 (2.5%)	6 (1.2%)	2 (0.4%)
Bosutinib	Pleural effusion	3 (1.4%)	8 (3.75%)	-
	Pulmonary arterial hypertension	2 (0.94%)	5 (2.35%)	-

In brackets, the percentage of ICSRs associated with dasatinib or bosutinib prescription.

**Table 5:** The reporting odds ratios (RORs) of serious Individual Case Safety Reports (ICRSs) associated with dasatinib and bosutinib prescription, signaled in the years 2020–2024 with the preferred terms “pleural effusion” and “pulmonary arterial hypertension” in the European Economic Area and the United Kingdom, compared with the signals associated with the prescription of the tyrosine kinase inhibitors asciminib, imatinib, nilotinib, and ponatinib. The total number of serious Individual Case Safety Reports (ICRSs) for dasatinib and bosutinib is 508 and 213, respectively.

Drug	Adverse Reaction	ICSRs (Cases)	All Other TKIs’ ICSRs (Cases)	ROR (95% C.I.)
Dasatinib	Pleural effusion	165	103	9.55 (7.28–12.52)
	Pulmonary arterial hypertension	34	31	4.90 (2.98–8.05)
Bosutinib	Pleural effusion	41	227	2.32 (1.61–3.36)
	Pulmonary arterial hypertension	18	47	4.70 (2.68–8.25)

Dasatinib or bosutinib cases were excluded from “all the other TKI ICSRs” when calculating each ROR. C.I. = confidence intervals.

## Data Availability

The data analyzed and presented in this study are available on the public EudraVigilance database, https://www.ema.europa.eu/en/human-regulatory-overview/research-development/pharmacovigilance-research-development/eudravigilance (accessed on 21 December 2025).

## Abbreviations

The following abbreviations are used in this manuscript:

TK	Tyrosine kinase
TKIs	Tyrosine kinase inhibitors
SARs	Suspected adverse reactions
FDA	Food and Drug Administration
EMA	European Medicines Agency
EU	European Union
UK	United Kingdom
EEA	European Economic Area
CML	Chronic myelogenous leukemia
BCR-ABL1	Breakpoint cluster region–Abelson 1
MedDRA	Medical Dictionary for Regulatory Activities
S/NS	Serious/non-serious ratio
PE	Pleural effusion
PAH	Pulmonary arterial hypertension
PT	Preferred term
FAERS	Food and Drug Administration Adverse Event Reporting System
ICSRs	Individual Case Safety Reports
SOC	System Organ Classification
ROR	Reporting odds ratio
N.A.	Not applicable
C.I.	Confidence intervals
